# Among the Lepers

**Published:** 1884-05

**Authors:** 


### AMONG THE LEPERS AT HONOLULU.

THE Hawaiian Kingdom, says a correspondent, has a leper population of two thousand. Of these, less than one-half are in custody. There is no physician on the island who knows enough about leprosy to convince any other physician that the truth has been reached. There are no white lepers under restraint, and probably not more than fifty or sixty whites are afflicted with the disease. There is no cure recorded in the rather meagre history of the disease. When a leper is reported to the police of any district in Hawaii an officer is sent to fetch him or her to Honolulu where there is a detention hospital put on the outskirts of the city and on the bank of the beautiful bay. Once declared a leper the person is civilly dead, and is incapable of suing in the courts or being sued. At the hospital there are accommodations for about one hundred and twenty persons.

The establishment consists of a plot of ground, say five acres, around which is a very high picket fence with a deep ditch outside of it. Within are cottages or wards. There is little shade except that made by the houses, no sward nor running water. The construction of the houses is primitive, and their furnishing plain to the verge of discomfort, but they are clean.

A steamer comes to take to the island of Molokai those whose condition is most advanced, there to remain until death. It was with such a party that I travelled to Molokai. It has been my lot to witness many sad scenes, but none of them approached in any way those which attended the separation of families, as this handful of lepers now sailed away to their exile.

Daughters reached their arms to mothers whom they might not embrace; wives held up their mouths for kisses which their husbands could not give; babes, held in arms of strangers, laughed and cooed to their mothers, to whose breaking hearts they might not be held in one last, loving c'asp. And sobs—such sobs, alas! that come from the depths of hearts wrung with the misery of a hopeless condition.

Presently the lines were cast off; the little steamer turned her head away and steamed slowly toward the bar. I went into the little cabin set apart for the captain, and closed the door, determined to hear no more and see no more of such grief The little port was open, when suddenly it was darkened, and, looking up, I saw the dark but beautiful face of a woman whose young husband was on his way to Molokai. She had swam out to intercept the steamer, and being—as, indeed, all her race are—as much at home on water as on land, she had no difficulty in accomplishing her purpose. "Ah," she said, "you are not a doctor nor a constable. Tell my husband to look over the side to me, and God will bless you."

I went on deck. We were steaming slowly, waiting for the government inspector to complete his task before taking his own boat for the shore. The lepers had become quiet, or comparitively so, except for pain. A few women were moaning, a young half white girl had flung herself on the deck in a wild abandonment of grief, and behind the smoke-stack I found the husband kneeling in prayer. His face, serrated with leprous sores, was held toward the sun; the tears were streaming down his cheeks and disease-cut features, softening them by their agony of supplication.

"William," I said, "your wife is alongside. Go quietly to the place that I shall point out to you, and you will see her." The man sprang up, and for a moment was perfectly beautiful, such a joy as came into his face. Then he turned and ran to the place I indicated. Half an hour afterward I saw him alone. We were then under a full head of steam, passing Diamond Head.

"Where is she?" I asked. He pointed astern, and there, not an eighth of a mile away, we saw her swimming toward some fishing boats, her soft, black hair floating out behind her, and her arm now and again waving to us good-by.

The leper settlement is generally called Molokai, but that, in fact is the name of the island upon which it is placed. Kalawai is the official name of the town or village. It is situated at the foot of a great ravine, through which at some time a stream of lava has poured. On either side are great precipices reaching into the sea. At their bases the surf beats high, sometimes beating as high up their brown hard fronts as seventy feet.

Here the fierce north-easterly trade winds roll up the seas they gather in a passage of two thousand miles over an unobstructed ocean. But the rocks stop them, and sullenly they fall back again with a heavy monotone. The two cliffs run backward in sharp ascents till they meet over the narrow pass through which admission to the leper settlement by land is alone possible. It is as if Nature had made a prison. One man could hold a thousand at bay over the bridle path, and one guard is sufficient to restrain the one thousand lepers which are at this place.

